# Phage Therapy in Prostatitis: Recent Prospects

**DOI:** 10.3389/fmicb.2018.01434

**Published:** 2018-06-29

**Authors:** Andrzej Górski, Ewa Jończyk-Matysiak, Marzanna Łusiak-Szelachowska, Ryszard Międzybrodzki, Beata Weber-Dąbrowska, Jan Borysowski, Sławomir Letkiewicz, Natalia Bagińska, Karen S. Sfanos

**Affiliations:** ^1^Bacteriophage Laboratory, Hirszfeld Institute of Immunology and Experimental Therapy, Polish Academy of Sciences, Wrocław, Poland; ^2^Phage Therapy Unit, Hirszfeld Institute of Immunology and Experimental Therapy, Polish Academy of Sciences, Wrocław, Poland; ^3^Department of Clinical Immunology, Transplantation Institute, Medical University of Warsaw, Warsaw, Poland; ^4^Medical Sciences Institute, Katowice School of Economics, Katowice, Poland; ^5^Department of Pathology, Johns Hopkins University, School of Medicine, Baltimore, MD, United States; ^6^Sidney Kimmel Comprehensive Cancer Center at Johns Hopkins, Johns Hopkins University, School of Medicine, Baltimore, MD, United States; ^7^Department of Urology, James Buchanan Brady Urological Institute, Johns Hopkins University, School of Medicine, Baltimore, MD, United States

**Keywords:** phages, prostatitis, inflammation, prostate cancer, phage therapy

## Abstract

Prostatitis has various etiology including bacterial infection and dysregulated immunity; some of its forms remain a serious therapeutic challenge. Inflammation occurs in all forms of this disorder and is proposed to predispose to the development of prostate cancer (PC). There are reports that phage therapy is effective in chronic bacterial prostatitis. Recent findings suggest that phages not only eliminate bacteria, but also mediate immunomodulating (for example, anti-inflammatory) functions. The immunomodulating effects of phages could be beneficial in treating all forms of prostatitis and play some role in the prevention of the development of PC. As the etiological factors contributing to the majority of prostatitis cases remains largely unknown, and management options are often likewise limited, phage therapy merits further research as an attractive therapeutic option given its immunomodulating effects irrespective of the underlying causative factor(s).

## Introduction

Prostatitis accounts for 25% of all office visits made to urological clinics and its effective treatment remains a challenge (Khan et al., 2017). 35–50% of men are affected by symptoms suggestive of prostatitis during their life time and the actual prevalence is approximately 8% (Rees et al., 2015). “Prostatitis" is heterogeneous syndrome and is categorized by the National Institutes of Health (NIH) consensus classification as chronic prostatitis/chronic pelvic pain syndrome (CPPS). CPPS is divided into four categories: (I) acute bacterial prostatitis; (II) chronic bacterial prostatitis; (III) chronic prostatitis/CPPS; and (IV) asymptomatic inflammatory prostatitis. Asymptomatic inflammatory prostatitis is defined as inflammatory infiltrates in prostatic tissue that are not recognizably associated with clinical symptoms (Krieger et al., 1999). Inflammation occurs in all categories of syndromes, although its mechanisms may vary.

Bacterial prostatitis is mostly associated with Gram-negative bacilli of the *Enterobacteriaceae* family, among which *Escherichia coli* predominates, as well as other bacteria such as *Pseudomonas aeruginosa*. More recent reports suggest a critical role of Gram-positive pathogens in CBP’s etiology. The most common are *Enterococcus faecalis* and *Staphylococcus aureus*. The role of coagulase-negative staphylococci and *Corynebacterium* sp., so far recognized as non-pathogenic, is also discussed (Letkiewicz et al., 2010). All those pathogens may be targeted by their specific phages.

The standard treatment for episodes of acute or chronic bacterial prostatitis is oral antimicrobial agents and typically fluoroquinolones or sulfamethoxazole and trimethoprim (Bactrim). Prolonged treatment (e.g., at least 6 weeks) is often used, and the choice of antibiotic must be consistent with agents that have good penetration into the prostatic tissue. Although the cure rate for acute bacterial prostatitis with oral antibiotics is high, a subset of men will experience recurrences (chronic bacterial prostatitis). Men who develop chronic bacterial prostatitis are again typically treated with repeat doses of oral antibiotics, and here the cure rate ranges from 0 to 90% depending on the drug used and the duration of treatment, and antibiotic resistance can occur (Stern and Schaeffer, 2000) (for more details, see Letkiewicz et al., 2010). Treatments beyond the use of antibiotics are limited for men with chronic bacterial prostatitis. The use of alpha blockers has been suggested to reduce recurrence, and surgical procedures including transurethral resection and even radical prostatectomy are sought in extreme instances (Stern and Schaeffer, 2000).

## Prostatitis and Inflammation

Although the prevalence of acute and chronic bacterial prostatitis as evidenced by the ability to culture bacteria from urine or prostatic fluid using standard microbiological methods is only 5–10% of prostatitis cases (de la Rosette et al., 1993), it remains a significant therapeutic dilemma because of poor penetration of antimicrobials (which may be aggravated by biofilm formation by microorganisms). Bacterial strains able to produce biofilms are hypothesized to be present in chronic bacterial prostatitis (Mazzoli, 2010). Additionally, prostatic corpora amylacea which are very commonly observed in the adult prostate as well as prostatic calcifications are hypothesized to be related to bacterial infections or biofilm-producing bacteria (Sfanos et al., 2009; Yanamandra et al., 2009; Mazzoli, 2010). Furthermore, increasing antibiotics resistance of pathogens is becoming a great challenge which calls for novel strategies in the prevention and management of the diseases (Wagenlehner et al., 2014).

The inflammation mechanisms involved in CPPS are poorly understood. Aside from bacterial prostatitis where an infectious agent can be identified by microbiological techniques, the etiological factors contributing to the majority of prostatitis cases remains unknown. Additional factors that have been proposed to contribute to CPPS include hormone abnormalities, neurogenic inflammation, and psychological factors such as stress, anxiety, and depression (PontarI and Ruggieri, 2004). The assessment of inflammation markers associated with CPPS is limited largely due to the lack of prostate tissue sample procurement as part of this condition. Analyses are limited to an indirect analysis of urine, expressed prostatic secretions (EPSs), and semial plasma. Assessment of these types of samples have pointed to inflammatory markers and mediators such as TNF-α, IL-1β, IL-6, INFγ, and IL-8, all of which are increased in cases versus controls (reviewed in PontarI et al., 2004).

Dysregulated inflammation in the form of autoimmunity against prostatic antigens has also been suggested in patients with CPPS and bacteria may act as the inciting factor in this scenario as well. Mast cells [which are resident in normal prostatic tissue (Sfanos et al., 2017)] may be key actors in the development of this syndrome (Breser et al., 2017; Pérez-Alvarado et al., 2017). Bacteria may activate mast cells via their TLR4 receptors (McCurdy et al., 2001; Kubo et al., 2007), while LPS induces NF kB in those cells (Nigo et al., 2006). Furthermore, IFN gamma has been shown to be crucial for the development of experimental autoimmune prostatitis and elevated levels of that cytokine were detected in the inflamed prostate of mice with prostatitis (Breser et al., 2017) as well as in expressed prostate fluid in patients with this syndrome (Ding et al., 2006).

## Inflammation and the Development of Prostate Cancer

The association of infection and/or inflammation of the prostate with prostate cancer (PC) has been suggested for some time. Roberts et al. (2004) have shown elevated relative odds of PC in men with history of acute or chronic prostatitis (CPPS was not associated at all in this study). The authors suggested further studies to assess the role of biochemical and tissue markers in the development of prostatitis. These types of studies are potentially confounded by detection bias as well as the fact that prostate inflammation occurs in many men without symptoms (reviewed in Sfanos et al., 2017). One new study potentially circumvented these problems by examining inflammation in biopsy samples taken from men without indication for biopsy, and determined that inflammation in benign tissues was positively associated with later development of PC (Platz et al., 2017). In a mouse model of cMYC driven PC, prostate infection with a human prostate isolate of *E. coli* accelerated cancer development and progression (Simons et al., 2015). Mouse models of prostatitis elicited by *E. coli* or *Propionibacterium acnes* infection have also demonstrated that chronic inflammation induces focal prostatic glandular atypia as well as other characteristic changes found in association human PC, such as reduced expression of the potential tumor suppressor Nkx3.1 (Elkahwaji et al., 2009; Khalili et al., 2010; Sinohara et al., 2013).

A recent review addressing the potentially pro-carcinogenic role of inflammation in PC development analyzed the predisposing mechanisms in detail (Sfanos et al., 2017). Normal prostates contain stromal and intraepithelial lymphocytes (the number of the latter cells is increased in patients with prostatitis). Macrophages and mast cells may also be detected while granulocytes are rare (their number increases in inflammation). Acute and chronic prostate inflammation occur in up to 90% of adult men with neutrophils prevailing in acute and mononuclear cells in chronic inflammation. Interestingly, the presence of tumor-infiltrating lymphocytes and macrophages is associated with bad while the presence of mast cells with good prognosis after radical prostatectomy. High number of inflammatory cells is believed to contribute to cancer development. One of the proposed mechanisms involves reactive oxygen species (ROS) released by those cells causing direct DNA damage and genetic instability. Other mechanisms responsible for inflammation-induced PC may also be involved including an increase in nuclear factor kappaB (NF-κB) signaling in inflammation-associated luminal cells in the epithelial cell fraction, stimulation of TLR4 on prostate epithelial cells by bacteria and its components leading to chronic inflammation with a subsequent promotion of tumorigenic events: cell proliferation, angiogenesis, metastasis, and immune suppression (Kang et al., 2012), reviewed in Sfanos et al. (2017). The authors hypothesize that epithelial cells of the prostate (PEC) may play similar role to that of intestinal epithelial cells (IECs) which act not only as a physical barrier but also as communicators to the underlying immune cells of gut-associated lymphoid system (GALT). Intra-urethral injection of LPS causes PEC hyperplasia, some inflammatory infiltration and production of pro-inflammatory cytokines IL-6, IL-16, and IL-17 by PEC (Dos Santos Gomes et al., 2017). Similar results were reported using uropathogenic *E. coli* instilled transurethrally into mice (Boehm et al., 2012). It has been demonstrated that PEC can express various TLR, CD14 and the adapter MyD88 (Mackern-Oberti et al., 2006). Acute infection increases TLR4 in rat prostate (Quintar et al., 2006). PEC may play a significant role in sustaining and amplifying inflammation though NF-κB activation and production of proinflammatory cytokines (Wong et al., 2009). In response to bacterial signaling human PEC can produce cytokines engaged in innate immune defense (Kim et al., 2014). *P. acnes* can induce secretion of cytokines and upregulation of their mRNA; this effect may facilitate colonization deeper into the prostatic tissue (Drott et al., 2010). Also, *Trichomonas vaginalis* induces pro-inflammatory cytokines in PEC through activation of ROS and NF-κB (Seo et al., 2014; Gu et al., 2016). PEC may also produce antibacterial chemokines (Linge et al., 2008). In addition, in experimental autoimmune prostatitis in rats, PEC acquire MHC class II antigens which suggests that PEC may also be antigen-presenting cells (Donadio and Depiante-Depaoli, 1997).

Of particular interest are the data demonstrating that inflammation can cause expansion of PEC progenitor cells. This expansion is dependent on IL-1R signaling: in IL-1R-null mice this process was reduced >50% relative to control (normal mice); interestingly, such mice still mounted a significant inflammatory reaction thus suggesting that the effect of IL-1R on inflammation-induced hyperplasia was at the epithelial–stromal interaction rather than in reducing infection (Wang et al., 2015). Those data confirm earlier reports that IL-1 plays a critical role in prostate development by activating insulin-like growth factor (IGF) signaling, a process also occurring during inflammatory reactive hyperplasia to elicit PEC proliferation (Jerde and Bushman, 2009). Moreover, platelet-derived growth factor may promote PEC proliferation (Wang et al., 2015). Interestingly, human prostate carcinoma cells express alphaIIb-beta3 integrin, originally reported to be expressed only in cells of megakaryocytic lineage (platelets). The presence of this integrin is associated with increased tumorigenicity, invasion, and metastases. Blockade of this receptor inhibits invasion of PC cells through a reconstituted basement membrane (Trikha et al., 1996). Furthermore, it may also inhibit tumor growth in bone thus offering a potential treatment of bone metastases (Sutherland et al., 2012).

The role of IL-6 has been implicated in prostate inflammation and IL-6 mRNA expression may be increased in areas of acute inflammation but not PC cells. However, application of anti-IL6 receptor antibody siltuximab has not provided clinical benefit in patients (Puhr et al., 2016). IL-6 has also anti-inflammatory properties (Gadient and Otten, 1997; Spooren et al., 2011). In addition, the final effect of IL-6 blockade may be dependent on the phase of tissue injury and repair promoting beneficial effects in the earlier case but inhibiting in later one (Prystaz et al., 2018). Especially, interesting are studies on the association between IL-10 polymorphism and PC risk. Most of those studies suggest that low-IL-10 expression genotypes are associated with increased PC susceptibility and potentially high-graded disease. However, those associations may also depend on patients populations, so further studies using different populations are needed (Puhr et al., 2016). IL-1 was found to be increased in prostatic secretions in men with chronic prostatitis (Nadler et al., 2000). Another argument for cancer-inducing properties of inflammation are the data suggesting that the use of non-steroidal anti-inflammatory drugs decreases the risk of PC; the effect is more pronounced in aggressive PC and in men with a history of prostatitis (Doat et al., 2017).

## The Potential Protective Role of Phage Therapy in Treatment of Prostatitis and Prevention of Cancer

In the face of increasing concern over antibiotic resistance phage therapy has recently been gaining increasing recognition and hope as a potential tool for combating untreatable infections (Lyon, 2017). Many reviews have recently been published addressing this issue in detail (Górski et al., 2016; Abedon, 2017; Brüssow, 2017; El-Shibiny and El-Sahhar, 2017; Lin et al., 2017; Pires et al., 2017). In our experience, good results may be achieved in up to 50% of patients treated, while failures may be caused by the development of phage resistance, superinfection with another pathogen, the appearance of high-titer phage-neutralizing antibodies and rarely side effects (Górski et al., 2016). Importantly, phages may penetrate biofilms and cause 90% reduction in their formation on catheters (Delcaru et al., 2016).

Data on treating prostatitis are very scarce in the available literature (Letkiewicz et al., 2010). For example, in their detailed in-depth review on phage therapy of human infections, Abedon et al. (2011) mention only one article from 1936 where the authors “had seen less relapse than with silver nitrate treatment.” Russian authors have reported eradication of infection in 80% of patients following intrarectal administration and a relapse rate of 64%. These results were similar to those achieved with fluoroquinolones, but fewer side effects were observed in patients treated with phages (Shormanov and Solovev, 2016).

Our preliminary data suggest that phage therapy could be efficient in patients with prostatitis. Our study comprised 27 patients most of whom received phages intrarectally for an average of 47 days. Eradication of pathogen as confirmed by two consecutive EPS cultures was observed in 13 patients. A significant decrease in the EPS leukocyte count, significant reduction of the prostate volume and an increase in the maximum urinary flow were also noted. No significant side effects were observed (Letkiewicz et al., 2009; Letkiewicz et al., 2010). Additional studies have also reported encouraging findings (Chanishvili, 2016). Optimal results have been achieved using intrarectal phage administration. No reliable proof of phage penetration into human prostate is available; however, in rats phage may penetrate prostate following intravenous administration (Letkiewicz et al., 2010).

**FIGURE 1:**
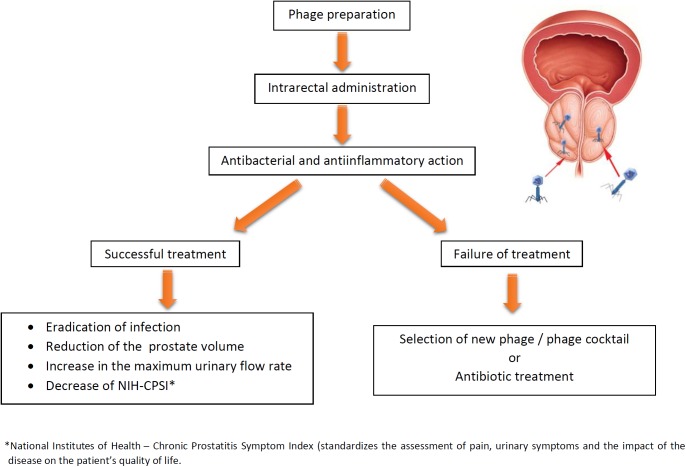
Potential and expected results of phage therapy of bacterial prostatitis. If the first round of therapy fails, the treatment can be repeated with another phage, phage cocktail, or antibiotics (phage therapy may reverse antibiotics resistance in bacteria).

**Figure 1** presents potential and outcomes of phage therapy in bacterial prostatitis. Furthermore, recent data indicate that phages are capable of crossing the epithelial cell layers from the gut, lung, liver, kidney, and brain (Nguyen et al., 2017), and therefore we propose that they should also cross PEC. It has recently been demonstrated that phages can use polysialic acid, a eukaryotic cell surface glycan involved in cell interactions, to bind and enter human neuroblastoma cells indicating “opening the door to eukaryotic cells” (Lehti et al., 2017). Moreover, phages could be armed with organ-specific peptides enabling their homing in target organs including prostate (Górski et al., 2015).

Furthermore, recent data indicate that – aside of their well-known antibacterial activity – phages may also cause immune modulating effects while their prevailing effect is anti-inflammatory. When considering confirmed phage activities with pathology of prostate inflammation it should be noted that phages have been repeatedly demonstrated to inhibit ROS production induced by bacteria and endotoxin, reduce NF-κB activation, diminish inflammatory infiltration induced by bacteria, endotoxin, and alloantigens and diminish platelet interactions with fibrinogen and platelet aggregation (Górski et al., 2017a). Phages can also diminish IL-6 production in later stages (7–10 days) of their interaction with bacteria (Sun et al., 2017). Phage protein may reduce IL-6 and inflammation in mice (Miernikiewicz et al., 2016). Moreover, phage therapy may normalize IL-6 production by patients’ cells *in vitro* inhibiting its high secretion (Weber-Dąbrowska et al., 2000). Phages can downregulate expression of IFNγ in human mononuclear cells (which appears to be especially important given its potential role in prostatitis) and upregulate the synthesis of IL-10 (which should decrease the development of PC), at least in some patients populations. In the era great interest in personalized medicine this option is of interest, especially in view of the fact that phage therapy itself is based on the principles of personalized medicine. Furthermore, phages downregulate TLR4 and MHC class expression (Van Belleghem et al., 2017) – both implicated in the immunopathology of prostatitis.

As mentioned earlier, the blockade of IL-1 receptor has been shown as an efficient means of prevention of inflammation-induced PEC hyperplasia. Interestingly, phages have been demonstrated to strongly upregulate the expression of the interleukin-1 receptor antagonist (Van Belleghem et al., 2017). Thus, it cannot be excluded that phage therapy in prostatitis may have multiple targets: (1) eradication of infection; (2) reduction of inflammation, and (3) control of excessive PEC proliferation, thus diminishing probability of the development of PC. In addition, there might be another mechanism by which phages may diminish the probability of PC by reducing inflammation and its direct action on PC. As already discussed, PC cells acquire the expression of integrin alphaIIb-beta3, relevant for tumor expansion and metastasis. We have found that T4 like phages express the KGD sequence (Lys-Gly-Asp) an analog of a well-known RGD sequence (Arg-Gly-Asp, being a target for integrins). KGD is also present within CD40 ligand; CD40–CD40L interactions stimulate inflammation and are relevant for T and B cell activation, while their interruption has anti-inflammatory and immunosuppressive properties [e.g., downregulating autoimmune reactions (Górski et al., 2015)]. Moreover, blockade on this integrin on cancer cells may be responsible for antitumor effects of T4 phages in experimental cancer models in mice (Budynek et al., 2010).

As mentioned, Sfanos et al. (2017) have recently hypothesized that epithelial cells of the prostate may have similar functions as IEC in the intestinal tract (communicating with the immune system and influencing its activities)). Our recent hypothesis suggests that phages may regulate IEC functions thereby contributing to maintaining an immune homeostasis in the gastrointestinal tract and opening perspectives for novel forms of immunotherapy based on their targeting (Górski et al., 2017b). Evidently, this hypothesis could also involve PEC as well: in addition to their anti-bacterial action phages could also target PEC and downregulate their expression of TLR4, NF-κB, ROS, and MHC class II antigens while upregulating IL-1R antagonist.

## Conclusion and Perspective

Recent data strongly suggest that massive amounts of phages cross epithelium each day (approximately 3 × 10^31^) and migrate to other tissues (Nguyen et al., 2017). This finding supports our hypothesis on phage translocation and resulting phage-mediated immunomodulation and contribution to maintenance immune homeostasis (Górski et al., 2006, 2015; Barr et al., 2013). The first randomized, placebo-controlled, double blind clinical trial ongoing at the National Center for Urology, Eliava Institute of Bacteriophages in Tbilisi, and Balgrist University Hospital in Zurich (Clinical Trials.gov Identifier: NCT 03140085) may throw more light on the potential of phages in prostatitis (Leitner et al., 2017). Further studies are needed to determine if phages may be beneficial in the treatment of prostatitis, prevention of PC, and perhaps adjunct therapy in PC.

## Author Contributions

AG drafted the main part of the manuscript. EJ-M, MŁ-S, RM, BW-D, JB, SL, NB, and KS contributed parts of the manuscript. All authors approved the manuscript.

## Conflict of Interest Statement

AG, RM, BW-D, and JB are co-inventors of patents owned by the Institute and covering phage preparations. The remaining authors declare that the research was conducted in the absence of any commercial or financial relationships that could be construed as a potential conflict of interest.
